# Highly Polymorphic Materials and Dissolution Behaviour: The Peculiar Case of Rifaximin

**DOI:** 10.3390/pharmaceutics15010053

**Published:** 2022-12-24

**Authors:** Annalisa Bianchera, Marino Nebuloni, Nicola Colombo, Davide Pirola, Ruggero Bettini

**Affiliations:** 1 Food and Drug Department, University of Parma, 43121 Parma, Italy; 2 Interdepartmental Centre Biopharmanet-Tec, University of Parma, 43124 Parma, Italy; 3 Redox S.R.L., 20900 Monza, Italy

**Keywords:** rifaximin, dissolution, pseudopolymorphs, supersaturation, kinetic solubility

## Abstract

Rifaximin is a locally acting antibiotic practically insoluble in water. It presents several crystal phases characterized by different degrees of hydration. The aim of this work is to investigate the dissolution behaviour of rifaximin α, β, and amorphous forms in relation to their relative thermodynamic stability to contribute to clarifying possible solvent- or humidity-mediated conversion patterns. Kinetic and intrinsic solubility were investigated along with particle size distribution, specific surface area, and external morphology. The solution and moisture mediated conversion from metastable α and amorphous forms to stable β form were elucidated by coupling intrinsic dissolution test with chemometric analysis as well as by dynamic vapour sorption measurements. The dissolution behaviour of the α form stems mainly from the transition to β form that occurs upon exposition to relative humidity higher than 40%. The α form converted more rapidly than the amorphous form due to the smaller supersaturation ratio. It can be concluded that, due to its marked tendency to transform into β form, the dissolution test for the α form, even if conducted according to compendial procedures, needs to be accompanied by a panel of further tests that allow to uniquely identify the solid phase under investigation.

## 1. Introduction

Polymorphism, the ability of a molecule to exist in more crystal structures, is critical for the bioavailability of many drugs [1], since polymorphic forms of a drug display different physico-chemical properties, such as, among many, solubility and stability. The fact that polymorphism and pseudopolymorphism of active pharmaceutical ingredients play a crucial role in their dissolution rate, is now well established [2,3,4].

Rifaximin (Figure S1 in Supplementary Materials) can be classified among highly (pseudo)polymorphic drugs, as at least eight crystal phases have been described so far (α, β, γ, δ, ε) [5,6] and (ζ, η, and ι) [7,8,9]. It is a broad-spectrum antibiotic drug belonging to the family of rifamycins that acts on bacterial DNA-dependent RNA-polymerase [10]. Being poorly water-soluble and poorly permeable through the intestinal mucosa, it is included in BCS class IV. In fact, the very low absorption in the human intestine is exploited for its local effect in the treatment of gastrointestinal diseases, such as traveller’s diarrhoea and irritable bowel syndrome, as well as in the management of hepatic encephalopathy; all these pathologies require a topical action upon oral administration, and absorption and systemic distribution are considered an undesirable side effect.

It has been reported that the crystallization of rifaximin in different conditions and solvents leads to different solvated forms [11], with α, β, and amorphous being the most represented forms, impacting in a different manner on the antimicrobial activity of the drug [12,13], as well as on its bioavailability [14]. The β and α forms contain between 3 and 4.5 and 0.5 and 1.5 water molecules in the formula unit, respectively [6]. The amorphous form is reported to have pharmacokinetic behaviour similar to that of the poorly crystalline polymorph γ, which is the most soluble of the crystal forms, all exhibiting systemic absorption [5].

In this respect, therefore, rifaximin represents a paramount example of the strict relationship between solid-state and biopharmaceutical properties.

The literature analysis affords the picture of a complex pattern of polymorphs, as clearly described by Viscomi and Braga [5,6]. In detail, the β form is obtained from wet rifaximin; when sprayed with water, α, γ, δ, and ε forms completely revert, with a different rate (faster for α, slower for γ), to the β form; their interconversion depends on the environmental conditions, in particular relative humidity. α and δ forms show the lowest water content but do not directly convert into one another.

Nonetheless, the conversion from one form to another is not straightforward and not yet fully understood, especially with respect to the role played by ambient humidity and aqueous dissolution media in this phenomenon.

Given these premises, the aim of this work is to investigate the dissolution behaviour of the most important solid phases of rifaximin, namely α, β, and amorphous, in relation to what is known about the relative thermodynamic stability and to contribute to clarifying any possible solvent- or humidity-mediated conversion pattern.

## 2. Materials and Methods

### 2.1. Materials

Rifaximin α form was produced by Zach System (Milan, Italy).

Rifaximin β form was obtained by wetting the rifaximin α form with water in a fluidized bed (Glatt GPCG2, air flow: 15–20 m^3^/h, air temperature: 60 °C, spray rate: 10 g/min) and drying until a final water content of about 8% *w*/*w*.

The preparation of amorphous rifaximin was carried out by solubilization of the rifaximin α in dichloromethane (DCM) and subsequent evaporation of the solvent in Rotavapor at 35 °C, 80 rpm. The flask containing the product was then placed in a vacuum oven at 40 °C, 10^−1^ bar, for one night. Subsequently, the powder was ground in a mortar and placed again in the vacuum oven, under the same conditions previously described, for 4 days. The residual content of DCM was analyzed by gas chromatography and resulted in ≤1% *w*/*w*.

The unambiguous identification of the three solid phases was carried out by X-ray diffraction on powder for comparison with the literature data [5,6] (Figure S2 in Supplementary Materials). To this end, the Cambridge Crystallographic Database was used to obtain the X-ray Powder Diffraction beamline of pure alfa and beta rifaximin. The diffraction pattern of each pure crystalline phase was obtained by downloading the data file related to the relevant crystal phase in “.cif “ format from the Cambridge Crystallographic Database (CCD). This file was processed using the Mercury 4.0.0 software (Cambridge Crystallographic Data Centre, Cambridge, UK), to compute the powder pattern of the forms of rifaximin. Subsequently, data have been converted into Excel format and compared to those obtained experimentally.

Four further batches of Rifaximin α form were also obtained from Zach System (Milan, Italy) (B1M1 and B2M1) and Sanofi Italia (Brindisi, Italy) (B2M3 and B2M4). The crystallographic purity of these batches was directly certified by the producers.

### 2.2. Methods

#### 2.2.1. Analytical Method, HPLC

An Agilent 1200 Series HPLC-UV (Agilent Technologies, Santa Clara, CA, USA) equipped with an end-capped C18 column, 0.25 m × 4.6 mm, 5 µm (Supelco, Bellefonte, PA, USA), was used. The liquid chromatography method used is that reported in the “Rifaximin” monograph of the Ph. Eur. 10. The method parameters are summarized below:

Injection volume 20 µL; flow 1.4 mL min^−1^; duration of the chromatographic run 10 min; column temperature 40 °C; wavelength of the detector 276 nm. The mobile phase consisted of a mixture of 37 volumes of an ammonium formate solution (3.16 g L^−1^) corrected with dilute ammonia to pH 7.2 and 63 volumes of a mixture of methanol and acetonitrile (50:50 *v*/*v*). The retention time of rifaximin was ≈5.6 min.

#### 2.2.2. System Suitability

Being a Pharmacopoeia method, the system suitability was verified by calculating the relative standard deviation (RSD), the retention factor (k), the number of theoretical plates (N), and the symmetry factor (As). The parameters were determined on the chromatographic plot of the standard solution. The reference solution (c) was prepared by dissolving, with the aid of an ultrasonic bath, approximately 40 mg exactly weighed of rifaximin W.S. with the solvent mixture (composed of 40% acetonitrile and 60% water) and making up the volume to 100 mL with the same mixture, in order to obtain a solution with a concentration of 0.4 mg mL^−1^.

One millilitre of solution c was diluted to 50 mL with the solvent mixture to obtain a solution of 8 µg mL^−1^ concentration.

Values of the measured parameters were: RSD = 0.11; specific Ph. Eur. < 0.73; k = 1.84; N = 5851; As = 1.2; specific Ph. Eur. 0.8–1.5.

The RSD value was also calculated on a series of solutions at different concentrations in the range 40–3.8 10^−2^ µg mL^−1^. The linearity of the response of the instrument as a function of the analyte concentration was verified in the same concentration range. The obtained value of the correlation coefficient (R^2^) was 0.9999.

The limit of detection and quantification values were 8.6 10^−3^ and 2.87 10^−2^ µg mL^−1^, respectively.

#### 2.2.3. Determination of the Kinetics Solubility

Rifaximin powders were sieved through a 250 µm net. Thereafter, 1 g of each powder was mixed with 20 g of glass beads (1 mm diameter) to assure a good powder dispersion and introduced in the dissolution vessel of a Sotax AT7 smart dissolution apparatus containing 750 mL of phosphate buffer at pH 6.8 (Ph. Eur.), thermostated at 20 ± 0.5 °C, setting a rotation speed of 100 rpm. The temperature of the bath was selected to better discriminate the dissolution behaviour of the three rifaximin solid phases tested. Amber vessels were used to protect the solution from light. Samples (2 mL) were withdrawn at predetermined time intervals and filtered (regenerated cellulose, 0.22 µm) before HPLC analysis. Samples deriving from the dissolution of α and β rifaximin were injected as such, whereas samples stemming from the dissolution of the amorphous form were diluted with the mobile phase 1:5 for the samples collected up to 15 min and from 50 to 120 min, 1:10 for the samples collected from 20 to 45 min, while samples collected after 120 min were injected as such.

The stability of rifaximin in phosphate buffer pH 6.8 at 20 °C for up to 7 days was checked by exploiting the capability of the adopted HPLC method to put into evidence possible degradation products. The test demonstrated a maximum reduction of <15% of the initial rifaximin concentration, that was considered acceptable for the kinetic solubility studies over extended times.

#### 2.2.4. Determination of the Intrinsic Dissolution Rate

The intrinsic dissolution rate assay was performed as reported in the monograph of the European Pharmacopoeia.

Non-disintegrating compacts with a diameter of 0.8 cm (area exposed to the dissolution medium = 0.5 cm^2^) were produced for each solid phase of rifaximin by compressing about 100 mg of exactly weighed substance in the matrix of the intrinsic dissolution apparatus, at a pressure of 4 tons for 5 min.

The matrix containing the powder compact was placed in a vessel of the dissolution apparatus (Varian 705 DS) and positioned so that the surface of the compact was 3.8 cm from the bottom of the vessel.

The dissolution test was conducted for 120 min using a volume of 250 mL of buffer at pH 6.8 (Ph. Eur.), in amber vessels thermostated at 37 ± 0.5 °C, setting a rotation speed of 100 rpm. Two millilitre samples were withdrawn and filtered (regenerated cellulose 0.45 µm) after 15, 30, 45, 60, and 120 min from the introduction of the matrix in the vessel. A 2 mL sample of “white” solution was also collected before inserting the matrix into the vessel.

The quantity of rifaximin dissolved at various times was quantified by HPLC.

#### 2.2.5. Scanning Electron Microscopy (SEM)

Images of the powder of four batches of α rifaximin were recorded with a SUPRA™ 40 FESEM (Zeiss, Germany) using a voltage of 1 kV.

Each sample was placed on a small piece of double-sided conductive tape that had been previously applied on a metallic sample holder. A weak nitrogen flow was used to remove the particles in excess. Images were recorded at magnification ranging from 300× to 5000×.

#### 2.2.6. Particle Size Distribution

The powders of four batches of rifaximin α form were characterized in terms of particle size distribution using laser diffraction (Mastersizer 2000, Malvern, UK). About 500 mg of powder were dispersed in the air at 2.5 bar pressure with a vibration rate of 50%. The instrument obscuration threshold was set at 6%. Data were expressed as volume diameter of 10th (D_v,10_), 50th (D_v,50_), and 90th (D_v,90_) percentile of the particle population and Span calculated as: (D_v,90_ − D_v,10_)/D_v,50_. All particle size measurements were carried out in triplicate.

#### 2.2.7. Determination of the Specific Surface Area (SSA) Using the BET Method

Measurements of Specific Surface Area by gas adsorption (BET single point method) were performed on the four batches of α rifaximin using the Flowsorb II 2300 instrument (Micromeritics, Norcross, GA, USA).

An accurately weighed amount of sample (approximately 0.6 g) was degassed for 1 h at 100 °C in a stream of inert gas (nitrogen/helium 30:70) and subsequently subjected to adsorption measurements of the same gas mixture in the presence of liquid nitrogen.

For each sample, six measurements were carried out.

#### 2.2.8. FT-IR Spectroscopy

FT-IR spectra were collected with a ReactIR 15 (Mettler Toledo, Columbus, OH, USA) equipped with a DS FiberConduit 9.5 mm probe with spectral range 1850–650 cm^−1^.

The dynamic solubility test was carried out in a three-neck 250 mL round bottom flask equipped with the FT-IR probe and mechanically stirred at 250 rpm (RW16 Basic overhead stirrer, Ika, Staufen im Breisgau, Germany). The flask was immersed in a thermostatic bath at 20 °C, then 150 mL of pH 6.8 phosphate buffer solution was added and left to equilibrate under stirring for 30 min. Subsequently, 200 mg of each rifaximin phase were accurately weighed and immediately poured into the flask through the third neck. The same port was also used to withdraw the suspension samples (2 mL) that were filtered through a regenerated cellulose 0.22 µm filter before HPLC analysis.

The tests at controlled humidity were carried out at ambient temperature utilizing a saturated solution of Na_2_HPO_4_, NaCl or KI (all provided by Sigma Aldrich, Darmstadt, Germany) to obtain 95, 75 and 70% relative humidity (RH), respectively. The experimental set-up is depicted in Figure S4 in the Supplementary Materials. Briefly, the saturated solutions of the three salts were loaded into the bottom part of the container by means of a syringe. To test the humidity of the ambient conditions, the sample was placed on the top of the probe.

#### 2.2.9. Dynamic Vapor Sorption, DVS

DVS studies were performed with an Aquadyne DVS-2 (Quantachrome Instruments, USA) using a gravimetric approach. The instrument was calibrated in 0–90% RH range at 25 °C with a certificated standard of microcrystalline cellulose (Microcrystalline cellulose for water sorption isotherm measurements, CRM n. 302, individual identification n. 0441, E.U. Bureau of reference). The balances of the instrument were calibrated at 25 °C, 50% RH using a 200 mg standard weight prior to the measurement of each rifaximin sample. The samples of rifaximin (about 30 mg) were analysed at 25 °C, measuring the water vapour sorption in the 5–90% RH range (step size = 5% RH). The transition from one step to the next occurred automatically when the rate of weight variation was lower than 0.001% min^−1^ and in any case not earlier than 30 min from the beginning of the step.

#### 2.2.10. X-ray Diffraction on Powder, PXRD

X-ray diffraction patterns on powder were recorded at room temperature with the instrument “ULTIMA IV” RIGAKU, laying the sample on a static sample holder. The goniometric radius (distance between the X-ray tube and the sample holder or, the equivalent, between the sample holder and the detector) was 285 mm. The X-ray tube had a copper target, with a current intensity of 40 mA and a voltage of 40 kV: the radiation, generated by the Cockcroft-Walton method, was constituted by Kα1 (1.540562 Å) and Kα2 (1.544398 Å). The analytical conditions were the following: scanning mode fixed time; 2θ range 3 ÷ 40 deg; sampling width 0.02 deg; scanning time 1 s/step; sample holder amorphous glass (depth 0.2 mm).

#### 2.2.11. Statistical Analysis

The statistical analysis was performed with Microsoft Office Excel 16.16.21 software assuming a significance level fixed at *p*-value = 0.05. Standard deviation was used to indicate experimental variability on experiments carried out at least in triplicate.

## 3. Results

**Figure 1:**
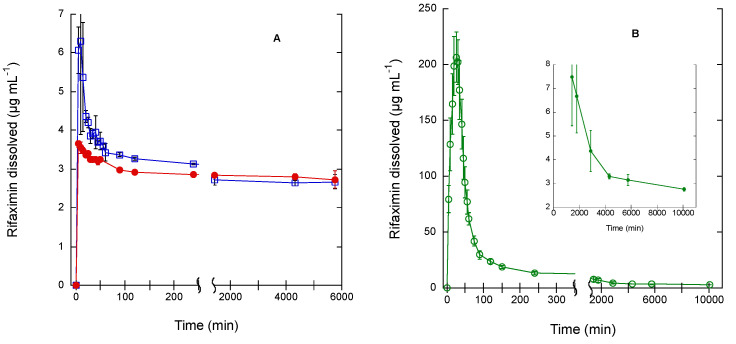
Concentration of rifaximin in solution at 20 °C from two crystalline phases (**A**); α form = empty square, β form = solid circle, and an amorphous phase (**B**) represented as a function of time. The insert in Panel B represent the data between 2000 and 10,000 min reported on a lower y scale. The bars represent the standard deviation (n = 3).

The kinetic solubility patterns in phosphate buffer at pH 6.8 of the three tested rifaximin forms are reported in Figure 1 as concentration vs. time. The relevant parameters are summarized in Table S1 in the Supplementary Materials.

As already reported, the β form represents the thermodynamically stable form in standard conditions and relative humidity >56%, while the other forms undergo a more or less rapid conversion [5,6]. Therefore, it was not surprising to observe that the β form afforded quite rapidly the lowest equilibrium solubility concentration of 2.73 ± 0.22 µg mL^−1^ after an initial, limited, and short supersaturation phase (maximum concentration 3.65 ± 0.09 µg mL^−1^), which may be ascribable to the presence of traces of amorphous rifaximin [5]. The alpha form afforded a significantly higher initial concentration (6.30 ± 2.43 µg mL^−1^), which was followed by a concentration decrease, slower than that observed for the β form, affording a final equilibrium value of 2.67 ± 0.19 µg mL^−1^. As expected, the amorphous form gave rise to a significantly higher initial concentration, reaching a maximum value of 206.50 ± 22.55 µg mL^−1^. The subsequent decrease was much slower than that observed for the α form (see insert in Figure 1B) approaching the equilibrium value of 2.72 ± 0.07 µg mL^−1^ only after 7 days.

**Figure 2:**
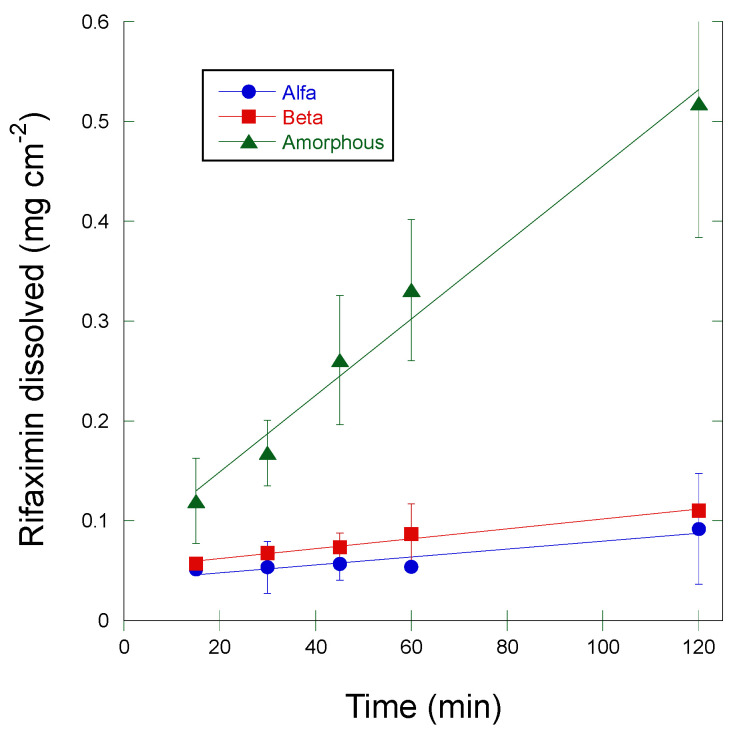
Amount of rifaximin dissolved at 37 °C per unit area from an amorphous phase (triangle) and two crystalline phases (α form, circle, and β form, square) represented as a function of time. The solid lines represent the linear regression of the experimental points. The bars represent the standard deviation (n = 3).

The amount of rifaximin dissolved per surface unit from the compacts prepared with the different solid phases is represented as a function of time in Figure 2. The linear regression of the experimental points between 15 and 120 min (linear portion of the dissolution curve) was performed to calculate the slope of the regression line which corresponds to the intrinsic dissolution rate expressed in mg cm^−2^ min^−1^. The values obtained are reported in Table 1 as mean value ± standard deviation.

The amorphous form gave rise to an IDR more than one order of magnitude higher than that of the crystalline phases. On the contrary, and quite curiously, rifaximin β form did not afford the lowest IDR, as could be expected from the relative thermodynamical stability [5]. However, the difference with the IDR with respect to the α form was not statistically significant (ANOVA, *p* > 0.05). However, it was observed that the compact of α form gave rise to a progressive disintegration at the solid-liquid interface rather than a uniform dissolution as the β and amorphous forms did. The constancy of the surface area of the compact is a crucial requisite in the IDR measurement. Thus, the observed phenomenon invalidates the reliability of the measurement as the solid liquid interface area did not remain constant. The experimental points relevant to the α form showed an upward curvature and in fact the R^2^ of the linear regression was only 0.87 vs. 0.98 for both the curves of β and amorphous form. The USP states that this type of curvature may be indicative of a systematic experimental problem such as compact delamination or disintegration.

**Figure 3:**
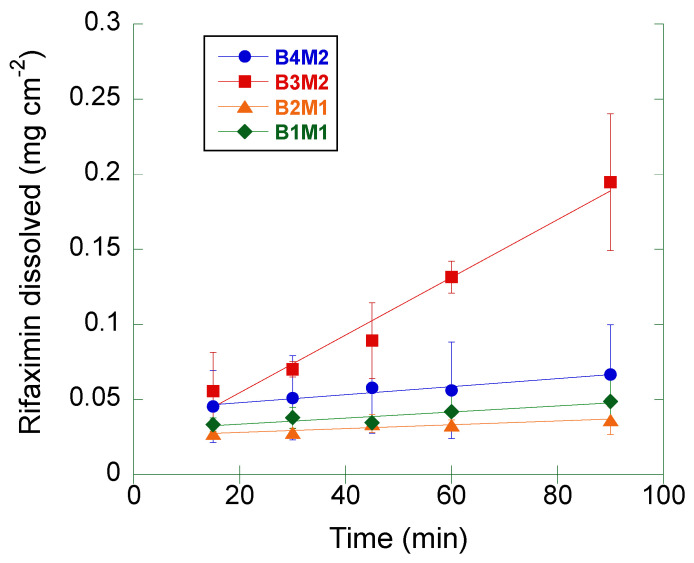
Amount of rifaximin dissolved at 37 °C per unit surface as a function of time from four different batches of rifaximin α form. The solid lines represent the linear regression of the experimental points. The bars represent the standard deviation (n = 3).

The same measurement was repeated on four different batches (from two different manufacturers) of crystalline rifaximin α form (Figure 3). The computed IDR values are summarized in Table 2.

The IDR of the samples corresponding to the batches B1M1, B2M1, and B4M2 was comprised between 1.2 and 2.7 mg cm^−2^ min^−1^, being the differences among these values not statistically significant (ANOVA, *p* > 0.2).

The IDR of batch B3M2 was significantly higher (19.2 mg cm^−2^ min^−1^) relative to the other α rifaximin samples (ANOVA, *p* < 0.02).

The IDR values determined in this second set of measurements were compared to that of the α form reported in Table 1: only the IDR of the batch B3M2 resulted to be significantly different (ANOVA, *p* < 0.05). Moreover, all batches, but the B3M2 one, gave rise to a non-significantly different IDR compared to that of the β form reported in Table 1.

Additionally, for all these measurements a progressive disintegration at the solid-liquid interface was observed. Thus, the same consideration made for data in Table 1 and Figure 1 applies.

**Figure 4:**
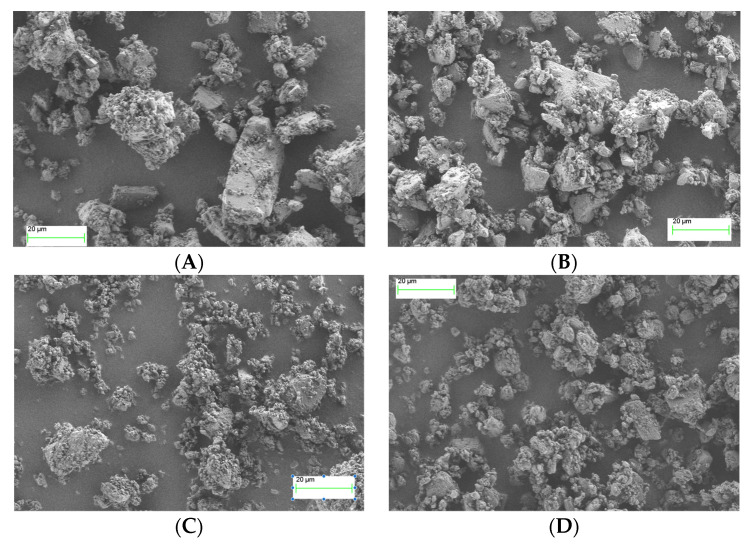
SEM pictures of samples of batch B1M1 (**A**), B2M1 (**B**), B3M2 (**C**), and B4M2 (**D**) taken at magnification 3000×.

The morphology of the particles of the four α rifaximin batches was investigated by scanning electron microscopy. As an example, pictures obtained at 3000× magnification for each powder are reported in Figure 4.

Particles of the batches B1M1 and B2M1 (Figure 4, panel A and B respectively) were very similar to each other being irregularly shaped and presenting a relatively large size distribution. Powders from the second manufacturer presented a different morphology; the particles of the batch B3M2 (panel C) were smoother with an apparently smaller size distribution compared to the particles of the batches of manufacture 1. In addition, besides some larger particles, the population contained a significant amount of very small particles. The fourth batch (B4M2, panel D) presented particles with a shape like that of batches B1M1 and B2M2 but smaller and more uniform in terms of size distribution.

The particle size distribution of these four batches was investigated in more detail by laser diffraction (Table 3).

As it can be appreciated, the laser diffraction data confirmed what was already anticipated by the simple SEM images observation, namely the high similarity between B1M1 and B2M1, and the smaller particle size of the batches B3M2 and B4M2, the latter presenting a slightly larger size distribution (span 2.13 vs. 1.9).

As the size distribution data did not fully correlate with IDR data, a further evaluation of whether the differences in observed IDR among the four α rifaximin batches could be ascribed to morphological differences noticed in the SEM experiments, specific surface area measurements were carried out with the BET method. The obtained results are reported in Table 4. The values of SSA scaled with the relevant IDR values, indicating that the observed IDR differences may be, at least in part, related to the different surface area stemming from the release of the single particles upon erosion of the compact. The largest surface area was presented by batch B3M2 which, however, was not the one with the smallest particle size distribution, suggesting that B3M2 could present a greater internal porosity of the particles compared to B4M2, which may justify the highest observed IDR.

**Figure 5:**
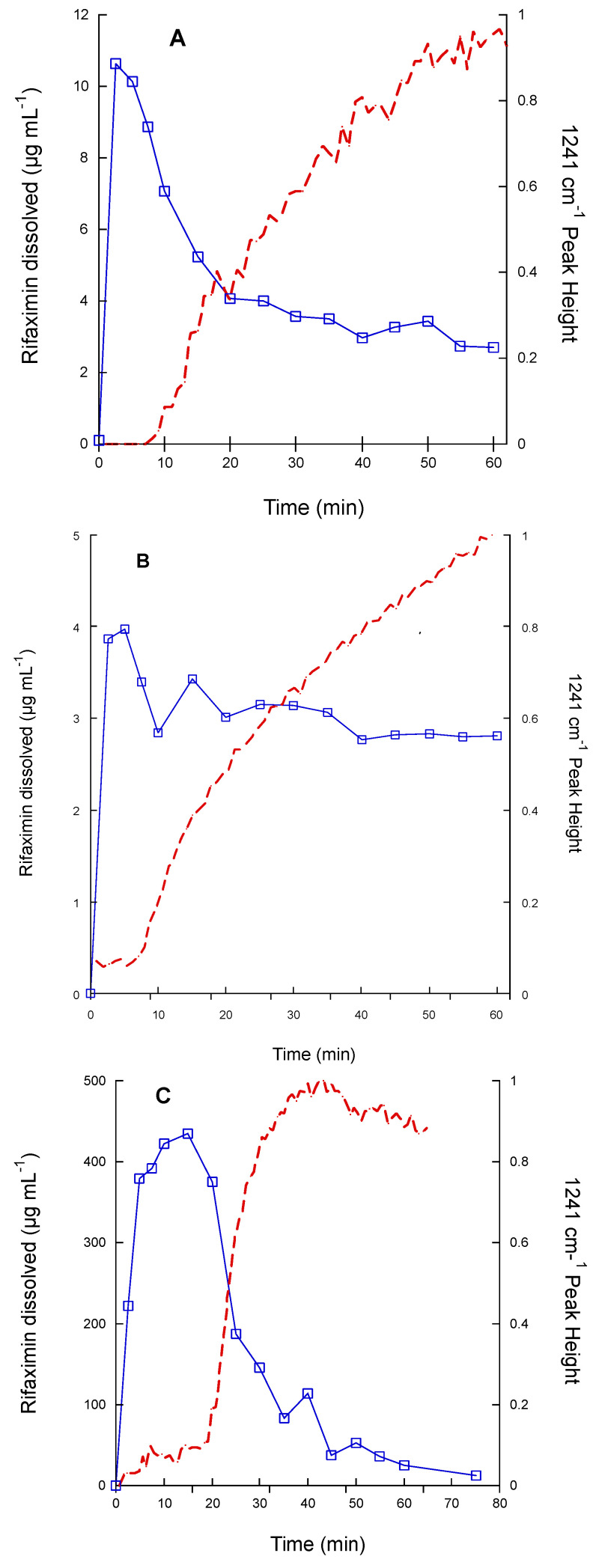
Concentration of rifaximin in solution at 20 °C (line with squares) from two crystalline phases ((**A**), α form; (**B**), β form) and an amorphous phase (**C**) along with the height of the ATR-FT-IR peak at 1241 cm^−1^ (dashed line) represented as a function of time.

Size and morphology variability of the α form is, however, not sufficient to explain the peculiar behavior reported in Figure 2 and, in some instances, also the variability of the data in Figure 3. Therefore, an in-depth investigation of the α -> β and amorphous -> β transitions in solution was carried out by coupling dissolution rate measurement by HPLC and solid-state monitoring with ATR-FT-IR. The FT-IR spectra of the three rifaximin forms are reported in Figure S3; two spectral regions were of interest for discriminating the three rifaximin forms: between 1570 and 1600 cm^−1^ and between 1220 and 1250 cm^−1^. In the second region, the peaks appeared more resolved, thus, diagnostic peaks were selected at the following wavenumber: 1227 cm^−1^ for the α form, 1241 and 1223 cm^−1^ for the β form, and 1230 cm^−1^ for the amorphous form. The dissolution test was carried out on 200 mg of rifaximin in 150 mL of phosphate buffer to improve the detectability. The obtained data are reported in Figure 5 as both drug concentration profile in solution and height of the IR peak at 1241 cm^−1^, as an indicator of the presence of β form in the suspended particles. In this experimental set-up, the α form (Figure 5A) attained a higher maximum value of concentration compared to the one reported in Figure 1A and reached the equilibrium concentration of the β form in about 1 h. The amorphous (Figure 5C) form as well afforded a significantly higher metastable solubility value compared to that of Figure 1B, while, despite the plateau reached by the IR signal relevant to the height of the 1241 cm^−1^ peak, the transition to β form apparently was not completed in 75 min, as indicated by the still relatively high value of concentration (11.7 µg mL^−1^). Nevertheless, for this form, as well as for the α form, the monitoring of the solid phase by IR spectroscopy, clearly indicated that the appearance of the diagnostic peak of the β form mirrored the kinetics of the dissolution curve. It is worth noting that the IR curve of the amorphous phase presented a larger initial lag time compared those of α and β forms. However, one can argue that the amount of rifaximin dissolved, especially in the case of the α form, does not seem to be sufficient to justify a complete (in the case of the α form) or almost complete (in the case of the amorphous form) solution mediated transition to β form as indicated by the values of rifaximin concentration recorded after 1 h.

**Figure 6:**
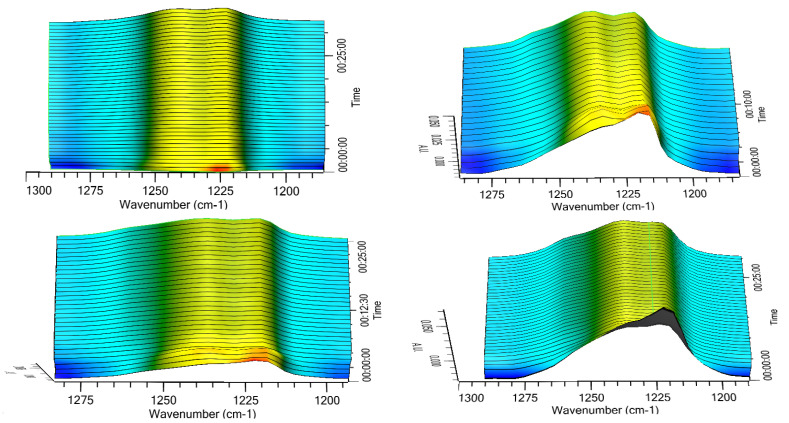
Expansion in the range 1300–1190 cm^−1^ of the 3D IR spectra of rifaximin α form exposed to environmental humidity (**upper left**), 70% RH (**upper right**), 75% RH (**lower left**), and 95% RH (**lower right**) evidencing the diagnostic peak of the α form (1227 cm^−1^) and β form (about 1223 and 1241 cm^−1^).

**Figure 7:**
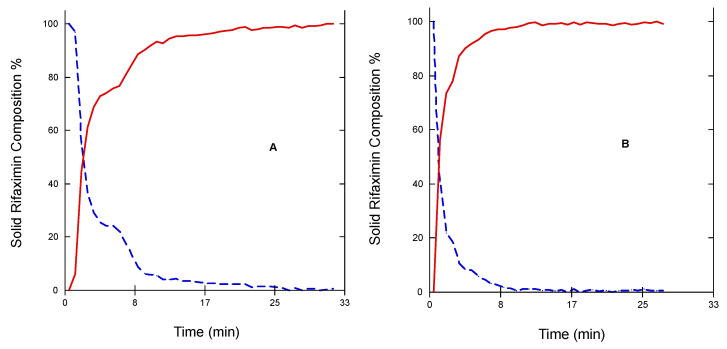
Trend upon chemometric analysis of the 3D IR spectrum of rifaximin α form exposed to environmental humidity (**A**) and 95% RH (**B**). Rifaximin α form (dashed line) β form (solid line).

Thus, the transition of the two metastable forms into the stable one was checked in a different experimental set-up, which implied the exposition of the three solid phases to room temperature and ambient humidity (64%), 70%, 75%, or 95% RH. The results relevant to the α form are reported in Figure 6 as 3D IR spectra in the 1300–1180 cm^−1^. It can be appreciated that the α form converted quite rapidly in all the tested humidity conditions. This conversion was practically complete after 15 min as indicated by disappearance of the peak at 1127 cm^−1^ and the attainment of a plateau of the height of the two peaks at 1223 and 1241 cm^−1^ (Figure 7) The two profiles of the two curves were perfectly specular, suggesting that the conversion from one form to the other occurred without formation of any intermediate form.

The same experiments carried out on the amorphous form did not afford any significant change of the diagnostic peak at 1230 cm^−1^ indicating that apparently the amorphous -> β transition does not occur upon exposure to humidity or at least not within the experimental timeframe adopted.

**Figure 8:**
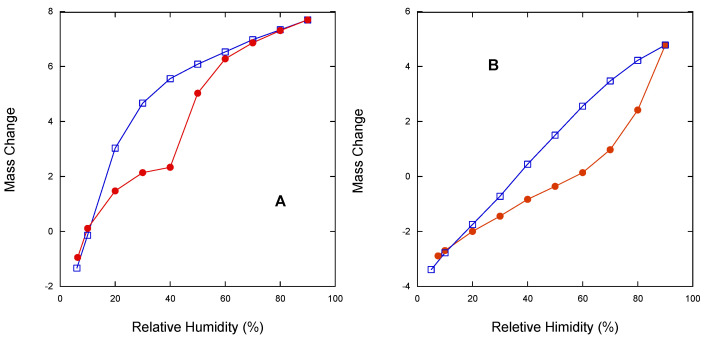
Isothermal Dynamic Vapor Sorption profiles at 25 °C of rifaximin α form (**A**), and amorphous rifaximin (**B**). Ascending curve solid circle, descending curve empty square.

Dynamic Vapor Sorption experiments were thus conducted to investigate in deeper detail the value of relative humidity at which the α -> β transition occurs. Figure 8 reports the DVS profile at 25 °C of α (Panel A), and amorphous rifaximin (Panel B).

Amorphous rifaximin presented a quite uniform weight gain as a function of RH with a limited change in slope that increased above 60% RH; the decreasing curve was smooth and almost linear. X-ray diffraction on powder recovered upon exposure to 70% RH still presented the typical halo of the amorphous material without any crystalline peak (see Supplementary Materials, Figure S5). Crystallization did not occur even after exposure to 95% R.H. for 20 days. Rifaximin α form afforded an ascending curve with a sharp slope change at 40% RH, while the descending curve decreased uniformly with a change in slope distributed between 40 and 20% RH. PXRD of the powder sample recovered upon stopping the measurement at RH > 40% clearly indicated the transformation in the β form (see Supplementary material, Figure S6A). The process was reversible as indicated by the fact that the PXRD pattern of the powder recovered in the descending curve at 10% RH was back to that of the α form (see Supplementary Materials, Figure S6B).

## 4. Discussion

Solubility is a general term for defining the maximum concentration that a compound (drug) can reach in a solvent at a given temperature. From the thermodynamical standpoint, it represents the equilibrium between the drug in the solid phase and the drug in solution. In general terms, therefore, the determination of the equilibrium solubility of metastable solid phased would be a paradox, being this, by definition, a non-equilibrium phase. In practice, the determination of pseudo-equilibrium solubility of metastable solid phases can be carried out provided that the energy barrier between the solid phase under investigation and its stable counterpart is sufficiently high to guarantee that the transition does not take place during the measurement time. In some cases, the solubility can be estimated by applying complex thermodynamical models [15]. In other cases, such as in the case of rifaximin, the determination of the so-called kinetic solubility is commonly used. This type of measurement offers, however, only a partial picture of the real solubility value of the metastable form, since the attainment of the maximum concentration value and the rate of decrease are largely depending on the test conditions, such as the ratio between the amount of solid phase and the solvent volume, as well as the stirring rate, as demonstrated by the discrepancy between data of Figure 1 and Figure 5. Data reported in Figure 1 clearly indicated that, irrespectively from the solid phase in the starting suspension, the concentration tended to a value of about 2.7 µg mL^−1^, which corresponds to the concentration of the solution in equilibrium with the rifaximin stable solid phase. Rifaximin α and β forms afforded very low values of maximum concentration compared to the amorphous phase, with α slightly higher than β, confirming that these crystal phases can be considered practically insoluble. These data are in good agreement with the literature [5,7] that reported C_max_ in solution slightly higher for α relative to β. This is somehow an obvious observation considering that the solubility in water of hydrate forms is lower than that of the relevant form at a lower degree of hydration, due to the fact that the relative stability of two pseudo-polymorphic phases does not depend only on temperature and pressure, as it happens for anhydrous phases, but also on the activity of the water [16]. The very low solubility of rifaximin α form implies a poor systemic absorption and is therefore strictly related to a favorable safety profile. In fact, pharmacokinetic studies both in dogs and humans have indicated that the absorption upon oral administration is less than 1% [5,11], while the fecal and urinary excretion represent 96.9 and 0.4%, respectively, of the administered dose [17]. On the other hand, the local antimicrobial efficacy is not separated from the presence in the intestinal lumen of a minimal and transient concentration of rifaximin in solution, which is guaranteed by the dissolution of the α form. From this point of view, the ability of the α form with respect to the β to give rise to an initial slightly higher, though not insignificant *oversaturation* is crucial.

Therefore, the capability of discriminating the dissolution behavior of the two forms represents a fundamental element in the formulation development and quality control of medicines containing rifaximin and intended to act locally in the intestine. The use of a compendial test such as the measurement of the IDR may be the solution to avoid the variability generated by the experimental conditions used in the kinetic solubility tests. However, data reported in Figure 2 and Table 1, as well as those in Figure 3, demonstrate that the intrinsic dissolution rate of rifaximin form α may be impossible to measure in a reliable manner, even in controlled and compendial test conditions. These phenomena can be considered responsible of the variability observed in Figure 3. The differences in morphology and particle size distribution shown in Figure 4 can only partly justify such variability. The thermodynamically driven α -> β transformation can be considered the main cause of the peculiar and variable behavior reported in Figure 2 and Figure 3. The disintegration of the compact at the solid-liquid interface that was already observed by Viscomi and collaborators [5] can be ascribed to the increase in the volume of the crystal cell due to the incorporation of water molecules stemming from the α -> β transition; in fact, as reported by Braga and collaborators, the volume of the cell can be estimated in 4445 and in 4580 Å^3^ for the α and β form, respectively [6]. The volume increase, in turn, determines the development of a disintegration force that breaks the compacts layers as already demonstrated for compacts of a poorly soluble compound such as nitrofurantoin [18]. As a further element supporting this interpretation, the data reported in Figure 2 shows a slightly positive deviation from linearity of the experimental points relevant to α form. In addition, for these points the recorded concentration values were lower than those of β form, although not in a statistically significant manner. According to USP, the positive deviation may be ascribed to a physical phenomenon such as disintegration, which was in fact observed. However, the compact disintegration led to an increase of the surface area exposed to the dissolution medium. Therefore, one should expect higher concentration values from α form relative to β form also considering the relative thermodynamic stability of the two crystalline forms. Again, referring to USP it must be remembered that for IDR experimental point also downward curvature of the dissolution profile may be observed because of a transformation of the solid form of the compact at the surface when a less thermodynamically stable crystalline form converts to a more stable form, such as in the case of the transition from an anhydrous form to a hydrate form. Therefore, one may speculate that the measured concentration values obtained from the intrinsic dissolution of the α form are the result of the superimposition of two phenomena, namely the disintegration, which leads to a progressive increase in the dissolution rate and the α -> β transition, which, on the contrary, reduces the dissolution rate over time. The result is that the data obtained could not be considered reliable and representative of the actual dissolution behaviour of the α form.

Real-time monitoring of solid-state during dissolution represents a tool to obtain a more accurate picture of the solution-mediated transition phenomena [19]; in this respect, the contemporary measurement of the amount of dissolved rifaximin and the appearance of the β form in suspension reported in Figure 5 and Figure 6 clearly evidence the transition mediated by the dissolution. However, the process appears to be not completed, likely due in part to the limit of the experimental set-up, which can detect only solid particles near to the probe, and in part to the complex process of dissolution/reprecipitation in β form.

In addition, DVS data offer a new element on what was already known on the interval of relative humidity at which the α form can be considered stable. Previous reports [5,6] indicated the limit of stability of β form > 56% RH; here, we show that the conversion α -> β occurs just above 40% RH while higher water activity is required to trigger the transition of the amorphous form.

Obviously, the transition from a less stable anhydrous (or less hydrated) metastable phase to the more stable and more hydrated stable one is thermodynamically driven by the presence of water or of a certain amount of humidity. However, the data relevant to the transition occurring upon humidity exposure (Figure 6 and Figure 7) offer further interesting elements to explain the articulated transition process that leads to the β form of rifaximin starting from its metastable solid phases and can be interpreted based on the thermodynamic and kinetic aspects discussed by Carstensen regarding the rate of conversion in moist storage [20]. In fact, it is well known that moisture is one of the main drivers for polymorphic conversion, and the mechanism behind this transition is the saturation of the moisture layer on the surface of the metastable solid phase which creates a supersaturated solution relative to the stable one that eventually nucleates leading the conversion. The conversion rate is a function of the nucleation rate, that according to the Classical Nucleation Theory [21], is inversely related to the supersaturation ratio, which represents the quotient of the concentration in solution and the concentration at the equilibrium. This ratio is high for poorly soluble compounds and represents a strong driver for the conversion. This consideration allows explaining both the observed very high conversion rate of the α form as well as the low tendency of the more soluble amorphous phase to transform into β form.

## 5. Conclusions

Among locally acting, poorly soluble compounds, rifaximin represents a paramount example of a combination of peculiar thermodynamic features that creates a complex pseudo-polymorphic profile that influences in a significant manner the observed dissolution behavior of the metastable forms. The commercially available medicinal product contains rifaximin α form, which, compared to other solid phases, presents a favorable combination of both toxicological and pharmacological profiles. Therefore, guaranteeing consistency between dissolution data and the crystal nature of the solid phase represents a crucial requisite for formulation development as well as for quality control. Here, we have demonstrated that the proper evaluation of the dissolution rate of this compound is highly challenging and should be approached with a clear awareness of the fact that α rifaximin is particularly unstable in contact with aqueous media or even in conditions of relative humidity higher than 40%.

We can conclude that, due to its marked tendency to transform into β form, the dissolution test for the α form, even if conducted according to compendial procedures, needs to be accompanied by a panel of further tests that allow to uniquely identify the solid phase under investigation.

## Figures and Tables

**Table 1:** Intrinsic Dissolution Rate (IDR) and coefficient of the linear regression of the experimental points (R^2^) of 2 crystalline forms and one amorphous form of rifaximin. Mean value ± standard deviation (n = 3).

Solid Phase	IDR mg cm^−2^ min^−1^ × 10^4^	R^2^
Alpha	3.95 ± 3.08 *	0.874
Beta	4.97 ± 3.20	0.979
Amorphous	38.28 ± 15.40	0.981

* Datum affected by compact disintegration.

**Table 2:** Intrinsic Dissolution Rate (IDR) and coefficient of the linear regression of the experimental points (R^2^) of the four α rifaximin samples. Mean value ± standard deviation (n = 3).

Batch Number	IDR mg cm^−2^ min^−1^ × 10^4^	R^2^
B1M1	1.99 ± 1.57	0.854
B2M1	1.24 ± 0.92	0.844
B3M2	19.19 ± 7.30	0.873
B4M2	2.67 ± 1.4	0.896

**Table 3:** Particle size distribution of the four α rifaximin samples expressed as volume diameter of the 10th, 50th, and 90th percentile of the distribution and span.

Batch Number	D_v10_	D_v50_	D_v90_	Span
B1M1	2.75	9.13	20.71	1.97
B2M1	2.87	8.80	19.47	1.89
B3M2	1.87	6.94	15.08	1.90
B4M2	0.92	4.89	11.34	2.13

**Table 4:** Specific Surface Area (SSA) of the four α rifaximin samples. Mean value ± standard deviation (n = 6).

Batch Number	SSA m^2^ g^−1^
B1M1	2.47 ± 0.18
B2M1	2.96 ± 0.15
B3M2	5.84 ± 0.57
B4M2	3.47 ± 0.05

## Data Availability

Not applicable.

## Supplementary Materials

The following supporting information can be downloaded at: https://www.mdpi.com/article/10.3390/pharmaceutics15010053/s1, Figure S1: Chemical structure of rifaximin; Figure S2: PXRD pattern of alfa (panel A), beta (Panel B), and amorphous rifaximin (Panel C); Curve n. 1 refers to the samples of this paper, curve n. 2 refers to the reference patterns from CCDC: alfa rifaximin (88651), beta CCDC (886514) [6]; Figure S3: FT-IR spectra of alfa (panel A), beta (Panel B), and amorphous rifaximin (Panel C); Figure S4: Experimental set up of the controlled humidity chamber. The arrow indicates the point where the samples were place on the probe window; Figure S5: PXRD of rifaximin obtained upon exposure of amorphous rifaximin to 70% RH in the DVS experiment; Figure S6: PXRD of rifaximin obtained upon exposure of α rifaximin to 50% RH (Panel A) and of the same powder recovered at 10% RH in the desorption curve (Panel B) in the DVS experiment; Table S1: Maximum concentration (C_max_), time to reach the maximum concentration (T_max_), area under the curve (AUC), and equilibrium concentration (EC) of the kinetic dissolution curves at 20 °C of two crystalline forms and one amorphous form of rifaximin. Mean value ± standard deviation. (n = 3).
