# Supplementary material for: Highly Polymorphic Materials and Dissolution Behaviour: The Peculiar Case of Rifaximin
Source: Pharmaceutics. 2022 Dec 24;15(1):53. doi: 10.3390/pharmaceutics15010053 (PMC9865978; doi:10.3390/pharmaceutics15010053)
Supplement: Supplementary file 1 [file pharmaceutics-15-00053-s001.zip › pharmaceutics-2101205-supplementary.pdf]

Supplementary Materials

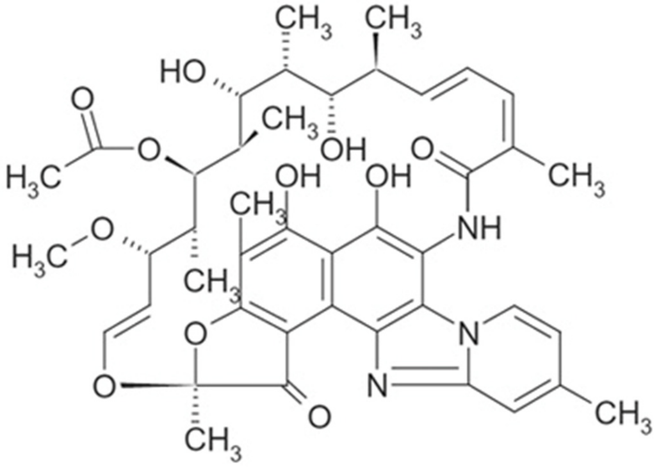

Figure S1. Chemical structure of rifaximin.

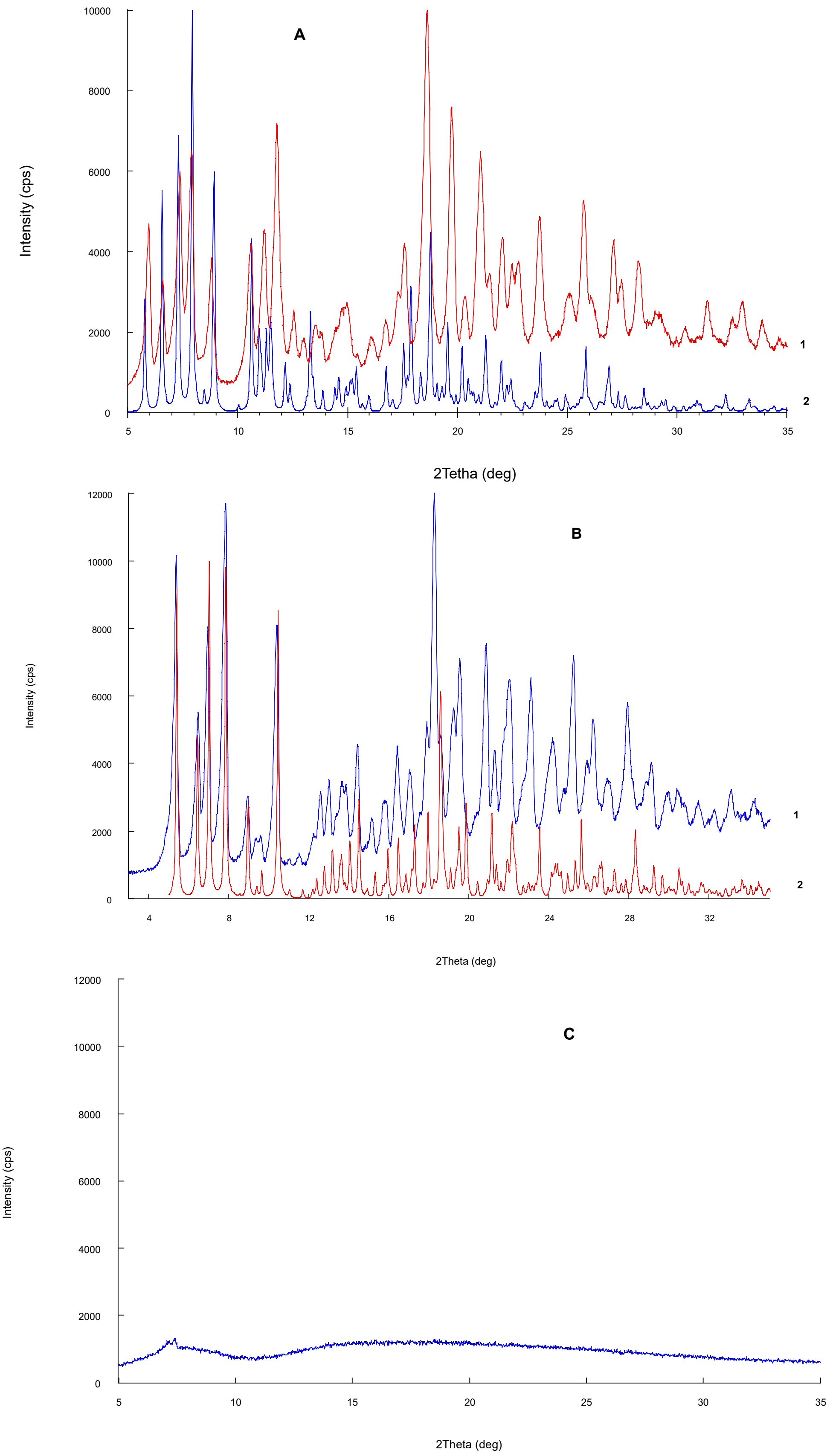

Figure S2. PXRD pattern of alfa (panel A), beta (Panel B) and amorphous rifaximin (Panel C); Curve n. 1 refers to the samples of this paper, curve n. 2 refers to the reference patterns from CCDC: alfa rifaximin (88651), beta CCDC (886514) [6].

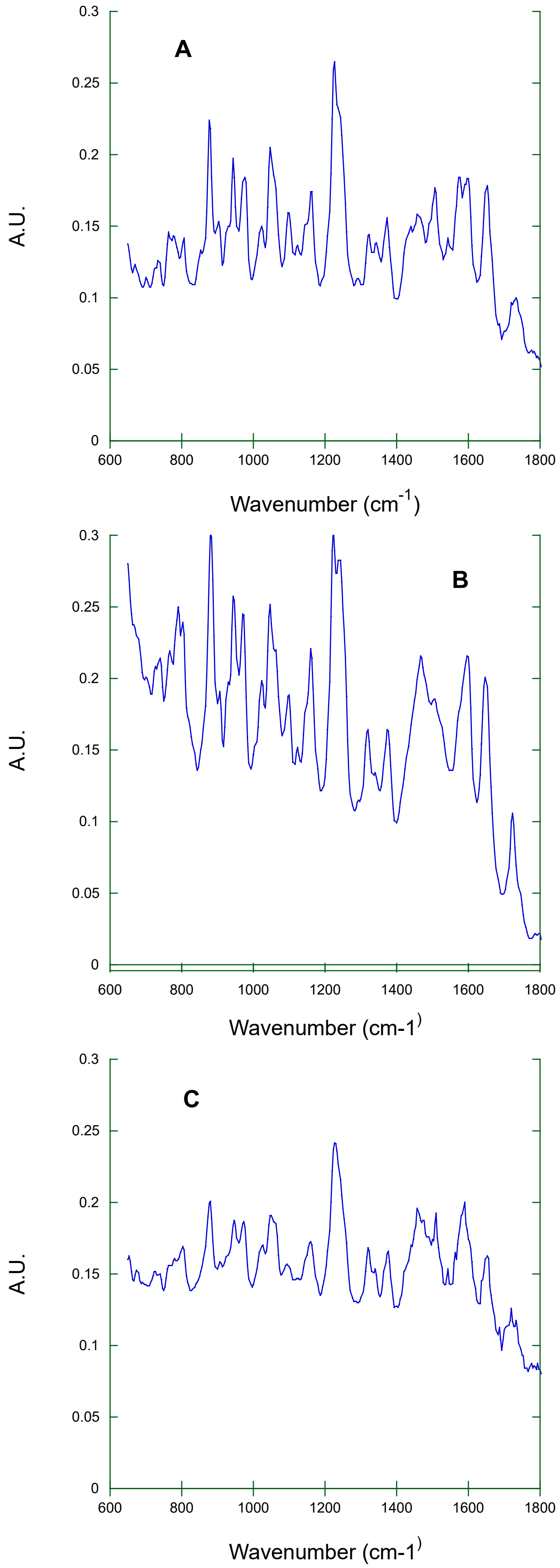

Figure S3. FT-IR spectra of alfa (panel A), beta (Panel B) and amorphous rifaximin (Panel C).

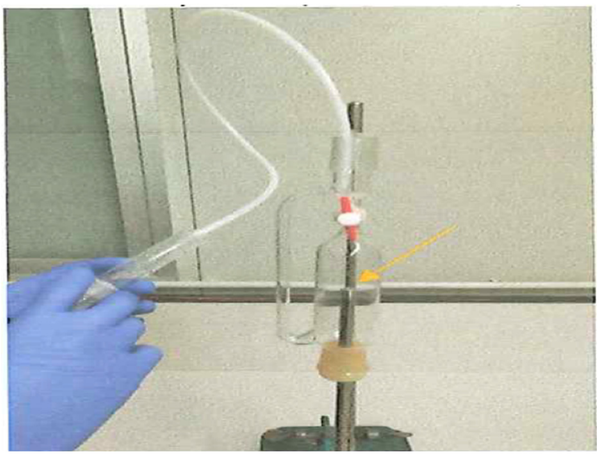

Figure S4. Experimental set up of the controlled humidity chamber. The arrow indicates the point where the samples were place on the probe window.

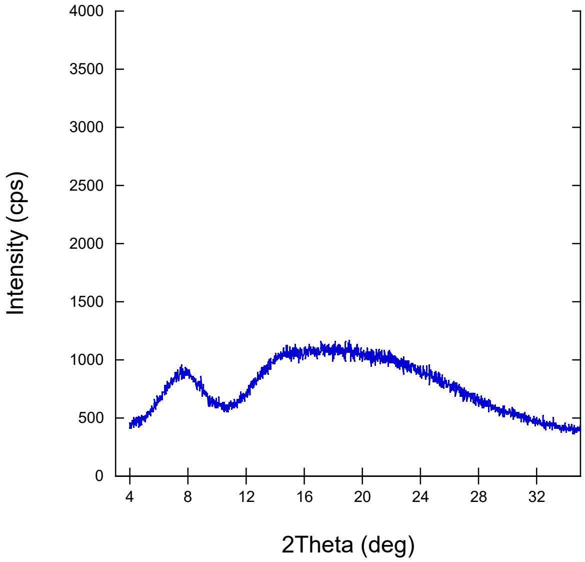

Figure S5. PXRD of rifaximin obtained upon exposure of amorphous rifaximin to 70% RH in the DVS experiment.

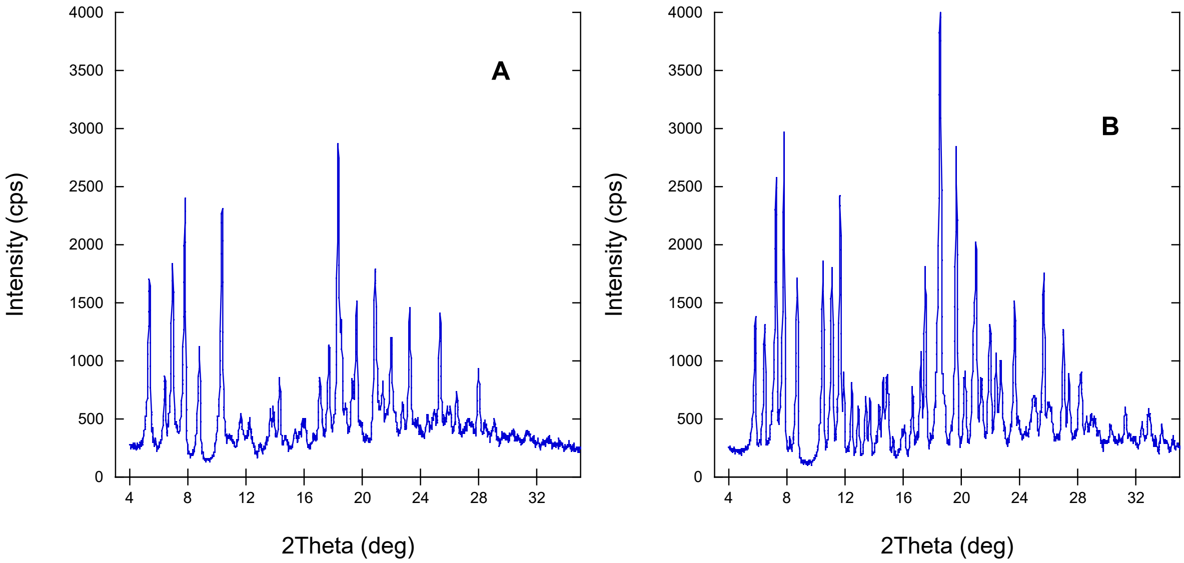

Figure S6. PXRD of rifaximin obtained upon exposure of *α* rifaximin to 50% RH (Panel A) and of the same powder recovered at 10% RH in the desorption curve (Panel B) in the DVS experiment.

Table S1. Maximum concentration (C_{max}) Time to reach the maximum concentration (T_{max}) area under the curve (AUC) and equilibrium concentration (EC) of the kinetic dissolution curves at 20 °C of 2 crystalline forms and one amorphous form of rifaximin. Mean value ± standard deviation. (n=3).

**Solid Phase**	**C_{max} μg mL^{-1}**	**T_{max} min**	**AUC μg min mL^{-1}**	**EC μg mL^{-1}**
Alpha	6.30 ± 0.19	10 ± 2	15893.1 ± 622.8	2.67 ± 0.19
Beta	3.65 ± 0.09	5 ± 1	16192.4 ± 437.2	2.73 ± 0.22
Amorphous	206.50 ± 22.55	25 ± 4	55986.2 ± 7602.7	2.72 ± 0.07
